# HECT ubiquitin ligases as accessory proteins of the plant proteasome

**DOI:** 10.1042/EBC20210064

**Published:** 2022-08-05

**Authors:** Zhishuo Wang, Steven H. Spoel

**Affiliations:** Institute of Molecular Plant Sciences, School of Biological Sciences, University of Edinburgh, Edinburgh EH9 3BF, United Kingdom

**Keywords:** HECT ligases, plant signal transduction, ubiquitin ligases, ubiquitin proteasome system, ubiquitin signalling

## Abstract

The proteasome plays vital roles in eukaryotic cells by orchestrating the regulated degradation of large repertoires of substrates involved in numerous biological processes. Proteasome dysfunction is associated with a wide variety of human pathologies and in plants severely affects growth, development and responses to stress. The activity of E3 ubiquitin ligases marks proteins fated for degradation with chains of the post-translational modifier, ubiquitin. Proteasomal processing of ubiquitinated substrates involves ubiquitin chain recognition, deubiquitination, ATP-mediated unfolding and translocation, and proteolytic digestion. This complex series of steps is made possible not only by the many specialised subunits of the 1.5 MDa proteasome complex but also by a range of accessory proteins that are recruited to the proteasome. A surprising class of accessory proteins are members of the HECT-type family of ubiquitin ligases that utilise a unique mechanism for post-translational attachment of ubiquitin to their substrates. So why do proteasomes that already contain all the necessary machinery to recognise ubiquitinated substrates, harbour HECT ligase activity? It is now clear that some ubiquitin ligases physically relay their substrates to proteasome-associated HECT ligases, which prevent substrate stalling at the proteasome. Moreover, HECT ligases ubiquitinate proteasome subunits, thereby modifying the proteasome’s ability to recognise substrates. They may therefore enable proteasomes to be both non-specific and extraordinarily selective in a complex substrate environment. Understanding the relationship between the proteasome and accessory HECT ligases will reveal how the proteasome controls so many diverse plant developmental and stress responses.

## Introduction

In eukaryotes, cellular degradation of proteins is predominantly regulated by the 26S proteasome, which functions as the major protease of the cytoplasm and nucleus. Proteins fated for degradation by the proteasome are marked post-translationally with ubiquitin. A well-characterised cascade of E1 activating, E2 conjugating and E3 ligase enzymes are responsible for the addition of ubiquitin to substrates destined for the proteasome. In this process, C-terminal glycine residues of ubiquitin are primarily attached to the ε-amino group of lysine residues in the substrate. Reiterations of this process can lead to attachment of additional ubiquitin moieties to one of seven internal lysine residues (K6, K11, K27 K29, K33, K48, K63) or the N-terminal Met1 of the preceding ubiquitin, thereby generating eight different ubiquitin chain topologies with diverse structural conformations [1,2]. Many of these topologies can be recognised by the proteasome but intrinsic ubiquitin receptors of the proteasome favour substrates with K48-linked chains [3–10].

In humans, proteasome dysfunction is the underlying case of many pathologies. Likewise, the ubiquitin-proteasome system plays vital roles in the growth and development of plants and their response to ever-changing environmental cues. Compared with animals, the genomes of higher plants encode for hundreds or thousands of components of the ubiquitin and proteasome signalling pathways, suggesting they play key roles in numerous cellular processes. Indeed, the ubiquitin-proteasome system is an intricate part of plant hormone signalling to orchestrate extensive transcriptional reprogramming. Many plant hormones directly or indirectly utilise ubiquitin E3 ligases to exert their effects by targeting transcriptional regulators for proteasome-mediated degradation [11–14]. In some cases, such as auxin- and jasmonic acid-mediated signalling, hormones act as a molecular glue that promote binding of Skp-Cullin-Fbox (SCF) E3 ligases to their substrates. As their substrates are transcriptional co-repressors, these SCF ligases act as nuclear hormone receptors that directly regulate the transcriptome. In case of the immune hormone salicylic acid (SA), its binding to substrate adaptors of Cullin3-RING Ligases (CRL3) may trigger conformational changes that control recruitment of its substrate NPR1, a transcriptional co-activator of immune genes [15–17]. Alternatively, other plant hormones indirectly activate E3 ligases to target transcriptional activators or repressors for degradation. Thus, the ubiquitin-proteasome system plays a key role in plant hormone signalling as well as many other signalling pathways.

Processing of ubiquitinated substrates by the proteasome involves an intricate series of sequential steps, including the recognition of the ubiquitin chain, removal and recycling of ubiquitin, engagement with the substrate, ATP-mediated polypeptide unfolding, and translocation into the proteolytic core where the substrate is cleaved into small peptides [18]. Recognition of ubiquitinated substrates is achieved through both intrinsic and extrinsic ubiquitin receptors that associate with the 19S regulatory particle of the proteasome. The presence of multiple diverse ubiquitin receptors may allow for several different ubiquitin chain topologies to be recognised by the proteasome. Rpn1, Rpn10 and Rpn13 function as intrinsic ubiquitin receptors that are located at the periphery of the proteasome complex and exhibit extensive flexibility to accommodate diverse ubiquitin conformations [7,19–22]. Spatial separation of Rpn10 and Rpn13 at the 19S regulatory particle may also explain why a chain of at least four ubiquitin molecules is required for the recognition of most substrates by the proteasome [21,23,24]. While Rpn10 shows preference for binding to distal ubiquitin, Rpn13 prefers proximal ubiquitin, thereby potentially providing a measure for ubiquitin chain length and architecture [25]. In contrast to intrinsic ubiquitin receptors, extrinsic receptors recognise substrates prior to their recruitment to the proteasome. Extrinsic receptors, such as Dsk2 and Rad23, recognise ubiquitin chains using ubiquitin-associated (UBA) domains, while docking with the proteasome via ubiquitin-like (Ubl) domains. These additional ubiquitin receptors likely increase the proteasome’s ability to recognise an even wider repertoire of substrates with complex ubiquitin chain conformations [26–28].

Once ubiquitinated substrates are recruited or delivered to the proteasome, their ubiquitin chain is cleaved or edited by the action of several deubiquitinases (DUBs), allowing ubiquitin to escape degradation and to be recycled. The JAMM metalloprotease Rpn11 is a key DUB located directly adjacent to the Rpn10 ubiquitin receptor. Proximity to Rpn10 allows Rpn11 to efficiently remove entire ubiquitin chains from substrates by cleaving the isopeptide bond between the first ubiquitin moiety and the substrate’s Lys residue [23,29,30]. In addition, Ubp6/Usp14 is a DUB and allosteric activator of the proteasome. Ubp6 interacts with the base of the 19S regulatory particle, where it stimulates ATPase activity of the proteasome and promotes opening of the 20S core particle gate to allow substrate access to the proteolytic chamber [18,31]. However, due to its proximity to Rpn11, Ubp6 can also obstruct ubiquitin binding to Rpn11 and interfere with proteasomal degradation [32]. These activating and inhibitory roles of Ubp6 make it an important allosteric regulator of the proteasome. The distinct DUB activity of Ubp6 cleaves ubiquitin chains *en bloc* with preference for substrates that contain multiple ubiquitin chains, suggesting Ubp6 may handle ubiquitin chains of higher complexities and either promote or excuse substrates from degradation [33]. Thus, Ubp6 allows the otherwise non-selective proteasome to become selective in choosing substrates for degradation. Finally, the proteasome recruits Uch37, which unlike Rpn11 and Ubp6, trims the ends of K6-, K11- and K48-linked ubiquitin chains, suggesting it debranches ubiquitin chains to promote substrate degradation [34–36]. Taken together, recruitment of ubiquitinated substrates and their engagement with the proteasome involves a complicated chain of events before the substrate is unfolded and threaded down into the proteolytic core complex [2,18,37]. Although insights into proteasome function were primarily obtained from yeast and animals, the widespread evolutionary conservation of proteasome subunits suggests that plant proteasomes function similarly.

To act as both a non-selective and highly specific cellular protease, the proteasome does not act in isolation but rather utilises a host of accessory proteins. In addition to the Ubl/UBA-containing extrinsic ubiquitin receptors and auxiliary DUBs described above, the proteasome regulatory particle physically interacts with ubiquitin ligases from various classes, including HECT (Homologous to E6-AP Carboxyl Terminus), RING, U-box, and SCF-type ubiquitin ligases [38–40]. The presence of multiple ubiquitin ligases and DUBs suggests that upon arrival at the proteasome, substrate-attached ubiquitin chains undergo considerable editing. The molecular and biological relevance of this ‘eleventh-hour’ remodelling of ubiquitin chains is now becoming clear and indicates that particularly HECT-type ligases play a key role in processive degradation of proteasome substrates.

## Structure and regulation of proteasome-associated HECT ligases

HECT ligases are a unique class of ubiquitin ligases that contain a C-terminal HECT domain that interacts with both an E2 enzyme and ubiquitin. The HECT domain utilises an active site Cys residue that forms a thioester bond with ubiquitin to transfer ubiquitin from the E2 onto the substrate. Consequently, unlike most E2–E3 enzyme complexes, the HECT domain can determine the specific ubiquitin linkage type that is added to substrates. Indeed, different HECT ligases have been reported to generate diverse ubiquitin topologies, including K11, K29, K48 and K63 linkages [41]. In addition, evolution of the HECT ligase family is characterised by extensive diversification of N-terminal protein–protein interaction domains [42,43]. The domain architectures of the N-termini are very diverse with some HECT ligases harbouring only a single protein–protein interaction domain, while others have several. These N-terminal domains are utilised to interact with substrates as well as with the proteasome. Through physical interaction, HECT ligases bestow proteasomes with ubiquitin ligase activity. Similar to extrinsic proteasome receptors, interaction of some HECT ligases with proteasomes is likely mediated by N-terminal UBA, Ubl and ubiquitin-interacting motif (UIM) domains. However, the Arabidopsis HECT-type UBIQUITIN PROTEIN LIGASE (UPL3) from the Ubiquitin Protein Ligase (UPL) family utilises an armadillo repeat domain-containing N-terminus to interact with proteasomes [44]. Arabidopsis UPL1, containing armadillo-type folds and an UBA domain, and UPL5, which harbours an Ubl domain, also interact with proteasomes [44,45]. In accordance with the various domain architectures employed for proteasome interaction, different HECT ligases utilise distinct docking sites on the 19S regulatory particle. While some HECT ligases interact with subunits Rpt4 and Rpt6 of the base ATPase ring and the Rpn1 ubiquitin receptor [39,46], Arabidopsis UPL3 interacts with the non-ATPase subunit Rpn7 of the lid complex [44]. It is conceivable that these distinct positions on the 19S regulatory particle may allow HECT ligases to engage with substrates and their ubiquitin chains in a variety of geometrical conformations, which could aid in establishing substrate selectivity of the proteasome.

The activity and function of HECT ligases is regulated by various mechanisms, including phosphorylation, intermolecular interactions, intrinsic catalytic activity-mediated ubiquitination, strength of E2-HECT domain interactions and interaction with adaptors and DUBs [47]. Many HECT ligases, including Arabidopsis UPL3 [44,48], show intra- or intermolecular interactions. In case of animal HUWE1 (HECT, UBA, and WWE domain containing E3 ubiquitin protein ligase 1), self-association leads to both intra- and intermolecular interactions that control its catalytic activity, with dimerisation suppressing its activity [49]. Moreover, HECT ligases employ interacting adaptors that influence their phosphorylation or ubiquitination, or alter their affinity for E2 enzymes [47]. Dynamic exchange of adaptor proteins may allow HECT ligases to fine-tune their activities, specificities and ubiquitin-chain topologies, making adaptors of particular interest for development of novel therapeutics.

## HECT ligases are key regulators of proteasome function

**Figure 1:**
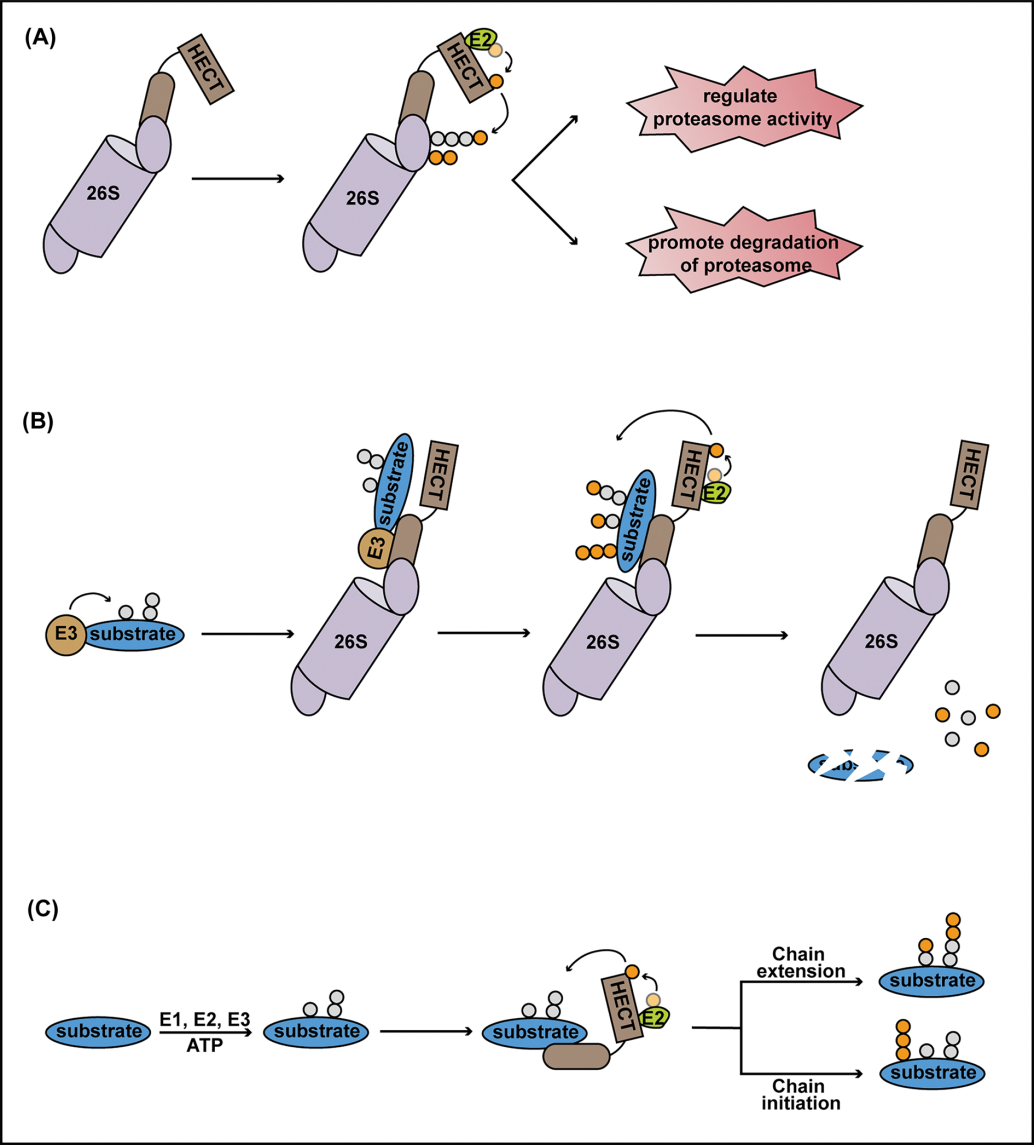
HECT ligases regulate proteasome function and processivity (**A**) HECT ligases ubiquitinate Rpn10, Rpn13 and possibly other components of the proteasomal 19S regulatory particle, thereby regulating substrate perception. HECT ligases also ubiquitinate 19S subunits of stalled or dysfunctional proteasomes to promote their autophagic degradation. (**B**) Pathway-specific E3 ligases (E3) relay ubiquitinated (grey circles) substrates to the proteasome by physical interaction with HECT ligases. This relay leads to HECT ligase-mediated ubiquitination (orange circles) of the substrate, which promotes its degradation by the proteasome. (**C**) HECT ligases either initiate the formation of new ubiquitin chains on the substrate or they elongate existing chains to promote substrate degradation.

Proteasomes receive ubiquitinated substrates for degradation, so it seems counterintuitive that they should also exhibit ubiquitin ligase activity. In this context, the role of proteasome-associated HECT ligases is probably twofold: (i) ubiquitination of proteasome subunits (Figure 1A) and (ii) further polyubiquitination of proteasome substrates (Figure 1B). At least 14 proteasome subunits and several proteasome-associated proteins have been found to be ubiquitinated [50]. In response to proteasome inhibition, heat shock and arsenite treatment, the mammalian HECT ligase Ube3c/Hul5 was found to ubiquitinate the ubiquitin receptor Rpn13 [50]. Rpn13 ubiquitination was reversible, suggesting it is a regulatory modification that controls proteasome function. Indeed, Rpn13 ubiquitination substantially decreased the binding of ubiquitinated substrates to the proteasome but did not affect core proteolytic activity. Thus, Ube3c/Hul5-mediated ubiquitination of Rpn13 may regulate the proteasome’s ability to receive substrates during periods of high proteolytic demand or prevent further substrate binding to stalled or defective proteasomes. Further regulation of substrate perception by the proteasome may be provided by modification of its ubiquitin receptor Rpn10. Yeast Hul5 and Rps5 as well as Arabidopsis UPL1, UPL3 and UPL5 have been reported to extensively ubiquitinate Rpn10 [44,51–53]. Monoubiquitination of Rpn10 inhibits its UIM domain and thus prevents it from interacting with ubiquitinated substrates [53]. Likewise, multi-ubiquitination of Rpn10 disrupts its interaction with the extrinsic ubiquitin receptors Dsk2 and Rad23 [54]. These findings suggest that during cellular conditions that induced proteolytic stress, HECT ligases fine-tune ubiquitin receptor availability for proteasome substrates (Figure 1A). Furthermore, yeast Hul5 was recently found to be one of three ubiquitin ligases to sequentially ubiquitinate dysfunctional proteasomes particularly on the 19S regulatory particle, resulting in their autophagic destruction (Figure 1A) [55].

In addition to modifying the proteasome itself, HECT ligases may help the proteasome to receive substrates and prepare them for degradation. In this context, proteasome-associated HECT ligases act as the final player in ubiquitin ligase relays that sequentially modify proteasome substrates. Recently, ubiquitin ligase relays of up to three ubiquitin ligases were reported in Arabidopsis [51]. Activity of the NPR1 transcriptional co-activator of plant immunity is precisely regulated by progressive ubiquitination and proteasome-mediated degradation. Initial ubiquitination of nuclear NPR1 by a modular CRL3 ligase facilitates NPR1 co-activator activity by stimulating its chromatin association at target promoters and leads to high expression of its target genes [17,56]. Subsequently, ubiquitin chains on NPR1 are elongated by the E4 ligase, UBE4, resulting in transcriptional inactivation of NPR1 and recruitment of the proteasome [56]. Upon arrival of the proteasome, NPR1 is further polyubiquitinated by the HECT ligases, UPL3 and UPL4, which is necessary for its proteasome-mediated clearance from target gene promoters [51]. Moreover, proteasome-mediated degradation of the ethylene-responsive transcription activator EIN3 is mediated by SCF^EBF1/2^ ligase, in which the EBF1 and EBF2 F-box adaptors specifically recruit EIN3 for ubiquitination [57–59]. Importantly, proteasome-associated UPL3 was found to physically interact with both EBF2 and ubiquitinated EIN3. Moreover, interaction between UPL3 and ubiquitinated EIN3 was dependent on EBF1/2 [51]. These findings suggest SCF^EBF1/2^ ligase physically relays ubiquitinated EIN3 to proteasome-associated UPL3 for further ubiquitin chain remodelling. As multiple E3 ligases have been reported to interact with the proteasome [39,40,60], relay of ubiquitinated substrates from pathway-specific E3 ligases to proteasome-associated HECT ligases may well be a general phenomenon (Figure 1B). In Arabidopsis, TIR1, COI1 and UFO F-box adaptors of auxin, jasmonate and floral signalling, respectively, were all found to associate with proteasomes [60]. Thus, it is plausible that many F-box adaptors from different SCF ligases relay their substrates to proteasome-associated UPL ligases for further ubiquitin chain remodelling and proteasome-mediated degradation. Curiously, the genome of apple encodes a HECT ligase, UPL7, that contains N-terminal Ubl and F-box domains [61]. It is conceivable that apple UPL7 directly incorporates into SCF ligases using its F-box domain, while associating with proteasomes via the Ubl domain.

**Figure 2:**
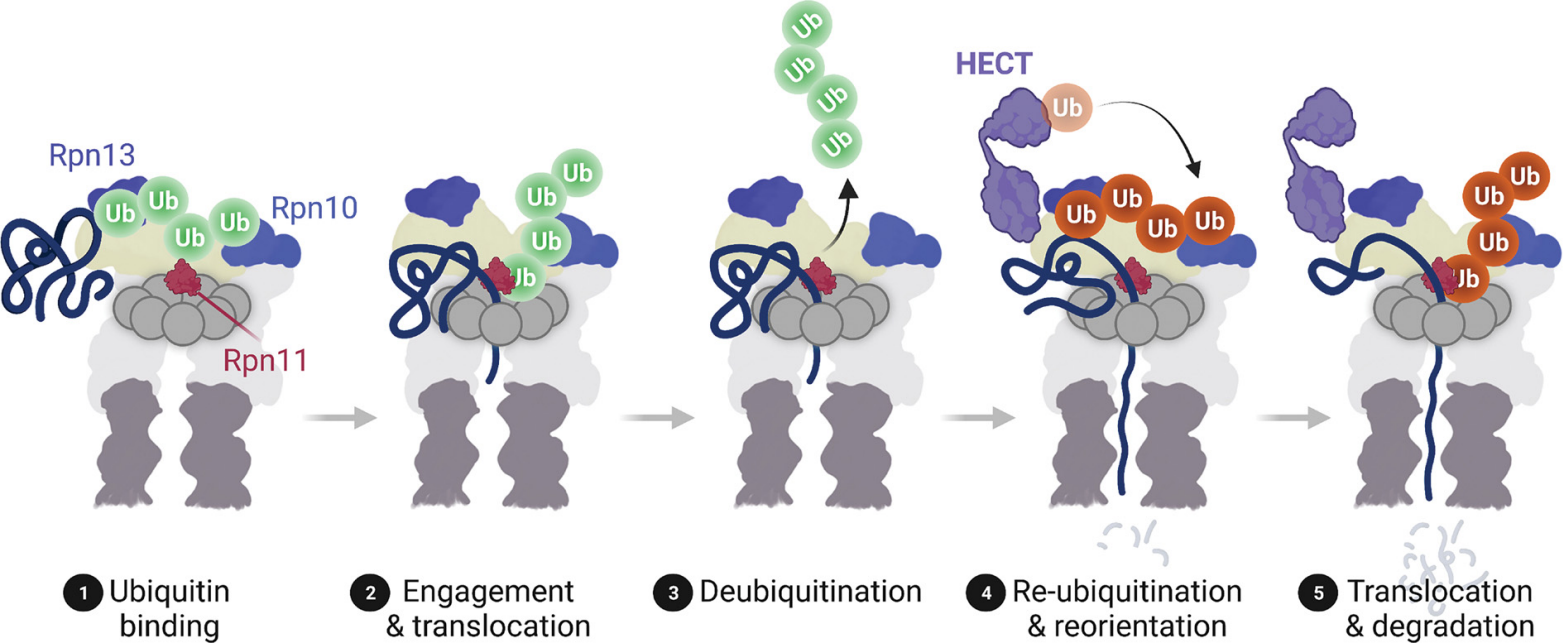
HECT ligases may promote proteasome processivity by geometrically reorientating substrates Ubiquitinated substrates are recognised by the proteasome through the Rpn10 and Rpn13 ubiquitin receptors (step 1). Subsequently, the proteasome engages the substrate, partially unfolds it and initiates translocation into the 20S proteolytic core particle (step 2). Translocation proceeds until the ubiquitin chain encounters the proteasomal DUB Rpn11 or other accessory DUBs (not shown), which deubiquitinate the substrate (step 3). Proteasome-associated HECT ligases re-ubiquitinate substrates (addition of red-coloured ubiquitin) to geometrically reorientate them for further unfolding and translocation, and to ensure they retain high affinity for proteasomal ubiquitin receptors (steps 4 and 5). Created with BioRender.com.

If numerous ubiquitin ligase relays end with proteasome-associated HECT ligases, it would suggest that unlike most substrate-specific E3 ligases, HECT ligases are far less specific and thus have much larger substrate repertoires. Indeed, single mutants of Arabidopsis *UPL1*, *UPL3*, *UPL4* or *UPL5* exhibit a substantial fall in the cellular level of ubiquitin conjugates [44,51]. In yeast too, Hul5 was found to ubiquitinate numerous unrelated substrates [52]. So why are ubiquitinated substates modified further by HECT ligases even though they have already arrived at the proteasome? Mutation of proteasome-associated HECT ligases, including yeast Hul5 and Ufd4, as well as Arabidopsis UPL3 and UPL4 reduces substrate ubiquitination and degradation, indicating that ‘eleventh hour’ polyubiquitination enhances the proteasome’s capacity to degrade substrates [51,52,62]. While some HECT ligases, such as Hul5, exhibit clear E4 ligase activity to elongate existing ubiquitin chains [52], it remains unclear if they also initiate the construction of new ubiquitin chains on substrates (Figure 1C). It is conceivable that a combination of these two activities constantly decorate substrates with ubiquitin to ensure they continue to exhibit high affinity for the proteasome while being unfolded and degraded (Figure 2). This is supported by data showing that the Arabidopsis EIN3 strongly accumulated at proteasomes that lack UPL3 and UPL4 HECT ligases, indicating EIN3 degradation had stalled at these proteasomes [51]. Furthermore, proteasomes lacking the yeast and human Hul5/Ube3C HECT ligase partially degraded artificial reporter substrates, indicating they prevent substrate stalling and promote proteasome processivity [63,64].

In context of proteasome processivity, interplay between HECT ligases and other proteasome accessory proteins may be critical for substrate degradation. For example, interactions between pathway-specific E3 ligases and HECT ligases may couple ubiquitin chain initiation and elongation to promote proteasome processivity. Such an effect was reported for yeast Ufd4 HECT ligase, which when complexed with the RING-type ligase Ubr1, produces longer substrate-anchored polyubiquitin chains [65]. Moreover, Hul5 HECT ligase cross-talks with the proteasome-associated DUB, Ubp6. This DUB directly opposes the E4 activity of Hul5 by trimming substrate-linked polyubiquitin chains [52]. Notably, binding of Hul5 to proteasomes is stabilised by the presence of Ubp6, suggesting that opposing ubiquitin chain extension and trimming activities are both required for substrate degradation. We propose that the interplay between HECT ligases, pathway-specific E3 ligases, DUBs and proteasomal ubiquitin receptors may regulate substrate positioning on the proteasome during degradation (Figure 2). Initiation and elongation of substrate-anchored ubiquitin chains combined with HECT ligase-mediated alterations in binding affinities of proteasomal ubiquitin receptors may allow the proteasome to continuously readjust the geometrical orientation of substrates to optimise their unfolding and/or threading into the proteolytic core. Failure to do so either leads to escape of the substrate, due to loss of affinity for the proteasome, or substrate stalling, leading to incomplete degradation. Alternatively, HECT ligases may allow the proteasome to become highly selective by inhibiting the affinities of proteasomal ubiquitin receptors or by promoting Ubp6-mediated *en bloc* deubiquitination, thereby excusing substrates from degradation.

## Proteasome-associated HECT ligases in plant cell signalling

In keeping with their impacts on proteasome function, mammalian HECT ligases are involved in a wide variety of cellular signalling pathways and their dysfunction underpins many pathologies similar to proteasome dysfunction [41]. Likewise, plant HECT ligases are regulators of diverse developmental and (a)biotic stress responses [48]. Numerous HECT ligase family members have now been identified in a variety of plant species, including various Brassicas, rice, soybean, wheat, tomato and apple [42,43,61,66–72], but only a few have known functional roles in plant cell signalling. Arabidopsis *upl3* mutants were initially identified as *kaktus-2* mutants due to abnormal development of trichomes that contain five or more branches rather than the three branches observed in wild-type plants [66,73]. UPL3 governs endo-reduplication cycles in trichomes, hypocotyls and cotyledons at least in part by targeting GLABROUS 3 (GL3) and ENHANCER OF GL3 (EGL3), two transcription activators of trichome development, for proteasome-mediated degradation [74,75]. UPL3 and its closest paralogue, UPL4, also regulate plant growth, development and seed set [44,76]. By aiding the proteasome in degradation of EIN3 activator, UPL3 and UPL4 regulate a variety developmental processes, including hypocotyl elongation, apical hook formation and root growth [51]. Moreover, UPL3 is required for the degradation of LEC2, a key transcriptional regulator of seed maturation, thereby controlling seed size and crop yields in *Brassica napus* [76]. Similarly, the HECT ligase UPL2/LARGE2 in rice was found to mediate the degradation of ABBERANT PANICLE ORGANISATION1 (APO1) and APO2, two positive regulators of panicle size and grain number [77]. Interestingly, APO1 is an orthologue of the F-box protein UFO that functions as an SCF ligase adaptor, while APO2 is an orthologue of the UFO target transcription factor, LEAFY (LFY). Therefore, it is plausible that in analogy to the SCF^EBF1/2^ – UPL3/4 ligase relay that targets EIN3 in Arabidopsis, APO1 relays APO2 to UPL2/LARGE2 for proteasome-mediated degradation in rice. Furthermore, Arabidopsis *upl3 upl4* double and *upl5* single mutants show accelerated leaf senescence, indicating they are negative regulators of senescence [44,78]. UPL5 exerts this effect by mediating the proteasomal degradation of the senescence-related transcription factor WRKY53 [78]. While it remains to be shown if all these developmental processes require UPL ligases to be associated with the proteasome, these ligases are clearly key regulators of proteasomal processes in plant development.

In addition to plant development, UPL ligases have been reported to play a key role in stress responses. Many *UPL* genes are responsive to a variety of abiotic and biotic stresses [61,69,72]. Indeed, mutant *upl3* and *upl4* plants were found to exhibit resistance to drought [79,80]. Moreover, UPL1, UPL3, UPL4 and UPL5 regulate disease resistance mediated by the plant immune hormone SA and consequently, the respective single mutants show severe susceptibility to the bacterial leaf pathogen *Pseudomonas syringae* [44]. These four UPLs are required for the SA-induced polyubiquitination and proteasome-mediated clearance of the transcriptional immune co-activator NPR1, and consequently mediate SA-induced transcriptional reprogramming [51]. While UPL3 and UPL4 act redundantly, if and to what extent they co-operate with UPL1 and UPL5 to promote SA signalling remains unknown. The importance of UPLs in regulating disease resistance is further underlined by a recent report showing that the effector GpRbp-1 from the potato cyst nematode *Globodera pallida* interacts with UPL3 [81]. Although it remains unclear how GpRbp-1 affects UPL3 function, it is likely that pathogen effectors inhibit UPL function akin to effectors that inhibit proteasome function [45]. Taken together, these findings suggest that UPL ligases may be promising targets for the improvement of developmental and stress response traits in plants.

## Perspectives

Studies of HECT ligases continue to transform our understanding of how the proteasome regulates substrate degradation and how it communicates with the signalling pathways that feed into it. Understanding how HECT ligases interact with other proteasomal accessory proteins to co-ordinate substrate degradation will likely require new structural insights. It is also likely that in response to their local environment, proteasomes may associate with different accessory proteins, resulting in distinct proteasomes with varying capabilities depending on cell type or subcellular localisation. In this context, different HECT ligases could not only play key roles in substrate degradation but they may also modify the pathway-specific E3 ligases they encounter during proteasomal substrate relays. HECT ligase-mediated ubiquitination of pathway-specific E3 ligases could provide the proteasome with a direct line of communication to specific cell signalling pathways in order to slow down or speed up substrate delivery.

The keen reader will have noticed that curiously, the majority of HECT ligase substrates identified in plants are key transcriptional regulators that control large parts of the genome. Indeed, UPL ligases were shown to govern transcriptional reprogramming of thousands of genes in response to ethylene as well as SA [44,51]. Specifically, a UPL3-proteasome complex was found to associate with ethylene- and SA-responsive promoters to facilitate the destruction of EIN3 and NPR1, respectively [51]. Thus, UPL3 and the proteasome effectively function as transcriptional cofactors to orchestrate the hormone-responsive transcriptome. Future proteomic studies may shed more light on the various transcriptional regulators and other substrates that UPL-proteasomes may control. A recent study that examined proteome-wide changes in the level of protein ubiquitination already highlights that UPL3 might indirectly altered the ubiquitination status of chromatin remodelling ATPases and histones [82], suggesting that UPLs may also be epigenetic regulators during transcription.

Taken together, future studies will likely reveal how HECT ligases work together with other accessory proteins to enable proteasomes to be both non-specific and extremely selective. Moreover, it may reveal how HECT ligases and the proteasome act together to feedback information into cell signalling pathways and how they specifically control many different plant developmental and stress responses.

## Summary

- HECT ligases are accessory proteins of the proteasome that promote proteasome processivity.
- HECT ligases ubiquitinate proteasome subunits and may help determine the proteasome’s balance between non-specificity and selectiveness for substrates.
- Ubiquitinated substrates are physically relayed from pathway-specific E3 ligases to proteasome-mediated HECT ligases to add additional ubiquitin chain complexity necessary for their processive degradation.
- Proteasome-associated HECT-type UPLs control the stabilities and activities of master transcriptional regulators of plant developmental and stress responses.

## Open Access

Open access for the present article was enabled by the participation of The University of Edinburgh in an all-inclusive *Read & Publish* agreement with Portland Press and the Biochemical Society under a transformative agreement with JISC.

## Abbreviations

APO1: ABBERANT PANICLE ORGANISATION1
CRL3: Cullin3-RING Ligases
DUBs: deubiquitinases
EGL3: ENHANCER OF GL3
GL3: GLABROUS 3
SA: salicylic acid
SCF: Skp-Cullin-Fbox
UBA: ubiquitin-associated
Ubl: ubiquitin-like
UIM: ubiquitin-interacting motif
UPL3: UBIQUITIN PROTEIN LIGASE 3
UPLs: Ubiquitin Protein Ligases
HECT: Homologous to E6-AP Carboxyl Terminus
HUWE1: HECT, UBA, and WWE domain containing E3 ubiquitin protein ligase 1
